# Oculomotor Profiles in Children Wearing Different Myopia Control Lenses: A Real-World Eye Tracking Study

**DOI:** 10.3390/vision10040067

**Published:** 2026-09-08

**Authors:** Shuo Wu, Hao Fu, Wenjin Li, Hao Li, Zehong Wang, Kai Wang

**Affiliations:** 1 Department of Ophthalmology, Peking University People’s Hospital, Beijing 100044, China; 2 Beijing Key Laboratory of Ocular Disease and Optometry Science, Peking University People’s Hospital, Bejing 100044, China; 3 Institute of Medical Technology, Peking University Health Science Center, Beijing 100044, China; 4 College of Optometry, Peking University Health Science Center, Beijing 100044, China

**Keywords:** myopia, pediatric myopia management, orthokeratology, defocus lens, diffusion optics technology, spherical equivalent, eye tracking, oculomotor behavior, binocular coordination

## Abstract

To compare oculomotor profiles among children wearing orthokeratology, defocus spectacle, and diffusion optics technology lenses in a routine pediatric myopia management setting. This real-world observational study included children who had worn one of the three lens types for approximately one year and had available eye tracking data. Lens type was selected during routine clinical care rather than assigned by the investigators. Oculomotor parameters were compared among lens groups using Kruskal–Wallis tests with false discovery rate correction. One-year spherical equivalent change was examined only in the defocus spectacle and diffusion optics technology groups. Adjusted and exploratory analyses included age, sex, baseline spherical equivalent, baseline axial length, axial elongation, and parental education categories. A total of 134 children were analyzed, including 46 in the orthokeratology group, 49 in the defocus spectacle group, and 39 in the diffusion optics technology group. Significant group differences were observed for microsaccade frequency (FDR adjusted *p* = 0.00099), peak velocity (FDR adjusted *p* = 0.00099), latency (FDR adjusted *p* = 0.00395), fixation disparity (FDR adjusted *p* = 0.00395), and conjugacy (FDR adjusted *p* = 0.0274). Saccade frequency and saccade amplitude did not differ significantly. One-year spherical equivalent change did not differ significantly between the defocus spectacle group and the diffusion optics technology group (−0.27 ± 0.43 D vs. −0.34 ± 0.36 D, *p* = 0.312). No statistically significant correlation was observed between spherical equivalent change and microsaccade frequency in the two spectacle lens groups (Spearman *r* = −0.231, *p* = 0.069). Parental education categories showed no consistent association with oculomotor parameters after correction for multiple comparisons. Children wearing different myopia control lenses showed selective differences in oculomotor profiles. Refractive change in the two spectacle lens groups was similar, and the observed oculomotor differences should not be interpreted as direct indicators of myopia control efficacy. Eye tracking may provide complementary information about visual behavior and lens adaptation, but prospective studies with age-balanced designs and longitudinal refractive and axial length outcomes are needed.

## 1. Introduction

Myopia has become a major public health concern in children and adolescents. Early onset and rapid progression are associated with a longer lifetime duration of myopia and a higher risk of high myopia and myopia-related ocular complications. A global systematic review estimated that the prevalence of myopia in children and adolescents has increased over recent decades and may affect more than 700 million children and adolescents by 2050 [1]. In China, recent cycloplegic refraction-based evidence also indicates a substantial burden of pediatric myopia, supporting the need for comprehensive and practical strategies for childhood myopia management [2].

Several optical and pharmacological interventions are used to slow childhood myopia progression. A recent living systematic review reported that multiple interventions can reduce spherical equivalent progression and axial elongation compared with inactive controls, although the magnitude and certainty of treatment effects vary across modalities [3]. International Myopia Institute reports further emphasize that myopia management should include refractive and biometric monitoring, visual function assessment, evaluation of the visual environment, adherence support, and communication with children and caregivers [4,5].

Orthokeratology lenses are worn overnight and temporarily reshape the anterior corneal surface, allowing daytime unaided vision while altering peripheral retinal defocus. Spectacle-based designs, including defocus spectacle lenses and diffusion optics technology lenses, are worn during daytime visual tasks and modify the retinal image through defocus, contrast modulation, or related optical mechanisms. Clinical studies have supported the efficacy of orthokeratology, defocus spectacle lenses, and diffusion optics technology spectacle lenses in slowing myopia progression in children [6,7,8,9,10]. Recent optical studies also indicate that myopia control spectacle designs can differ in their effects on retinal image contrast and peripheral image formation [10,11].

Most clinical studies of myopia control lenses have focused on spherical equivalent refraction and axial length. However, different lens designs may also influence visual quality, fixation behavior, saccadic timing, and binocular coordination during habitual viewing. Eye tracking provides an objective method for describing oculomotor behavior, including fixation duration, microsaccades, saccade frequency and amplitude, peak velocity, latency, and binocular coordination-related parameters. These measures are not established surrogate endpoints for myopia progression. Rather, they may provide complementary information about visual behavior and adaptation during lens wear. In addition, digital smart device use has been associated with myopia in children and young people, which provides further context for studying visual behavior during pediatric myopia management [12].

Age is particularly important when oculomotor parameters are interpreted in pediatric samples. Previous developmental eye movement studies have shown that saccadic latency tends to decrease with age during childhood, whereas peak velocity is more closely related to saccade amplitude and may be less dependent on age in some settings [13,14,15]. Microsaccades are small involuntary eye movements during fixation and have been linked to fixation control, visual sampling, and attention-related processes [16,17,18]. These findings suggest that age, task condition, and sensory input should be considered when oculomotor differences are compared across pediatric groups.

Parental factors may influence pediatric myopia management through treatment selection, adherence, outdoor activity, near work behavior, and follow-up regularity. However, a direct relationship between parental education style and short-term oculomotor parameters has not been established. Therefore, parental education categories were retained only as exploratory secondary variables in the present analysis, rather than as a primary focus.

The primary aim of this study was to compare oculomotor profiles among children who had worn orthokeratology, defocus spectacle, or diffusion optics technology lenses for approximately one year in a routine clinical setting. Secondary aims were to examine whether observed oculomotor differences were influenced by age and ocular covariates and to explore whether oculomotor parameters were associated with axial elongation or one-year spherical equivalent change. Spherical equivalent change was examined only in the two spectacle lens groups because post treatment refraction after orthokeratology may be influenced by corneal reshaping.

## 2. Materials and Methods

### 2.1. Study Design and Participants

This was a real-world observational study of children undergoing myopia management with orthokeratology lenses, defocus spectacle lenses, or diffusion optics technology spectacle lenses at a single tertiary ophthalmology center in China. The term “real world” refers to the noninterventional design, the use of routine clinical records, and the fact that lens type was selected during routine clinical care according to clinical indications, visual needs, parental preference, and shared decision making between clinicians and families. It does not imply national or multinational representativeness.

Children were included if they had completed approximately one year of lens wear and had usable binocular eye tracking data. The primary analysis focused on oculomotor profiles across lens groups. Axial elongation was examined as an exploratory secondary outcome. One-year spherical equivalent change was additionally evaluated in the defocus spectacle and diffusion optics technology groups, in which post-treatment refraction was not expected to be confounded by orthokeratology-induced corneal reshaping. The study was approved by the Ethics Review Committee of Peking University People’s Hospital (approval number: 2026PHB347-001; approval date: 21 April 2026). Written informed consent was obtained from parents or legal guardians, and assent was obtained from children when appropriate. The study was conducted in accordance with the Declaration of Helsinki.

### 2.2. Sample Size Rationale

No formal a priori sample size calculation was performed because this was an exploratory real-world observational study using available clinical and eye tracking records. All eligible children with approximately one year of lens wear and available eye tracking data during the study period were included. Therefore, the sample size was determined by data availability. This issue was considered when interpreting exploratory and multiplicity-adjusted findings.

### 2.3. Clinical and Baseline Variables

Age, sex, lens group, baseline spherical equivalent refraction, baseline axial length, one-year axial length, and available one-year spherical equivalent refraction were extracted from the clinical record. Baseline spherical equivalent refraction and baseline axial length were obtained at the pretreatment clinical visit before initiation of orthokeratology, defocus spectacle, or diffusion optics technology lens wear. For the orthokeratology group, baseline refraction was recorded before the start of lens wear to avoid the influence of corneal reshaping on baseline refractive status. Spherical equivalent was calculated as sphere plus half cylinder when necessary. Mean baseline spherical equivalent, mean baseline axial length, mean one-year axial length, and mean one-year spherical equivalent were calculated using available right and left eye data.

Axial elongation was calculated as mean one-year axial length minus mean baseline axial length. One-year spherical equivalent change was calculated as mean one-year spherical equivalent minus mean baseline spherical equivalent. Negative spherical equivalent change indicated greater myopic shift. Because orthokeratology changes corneal curvature and alters manifest refractive findings after treatment, spherical equivalent change was compared only between the defocus spectacle and diffusion optics technology groups.

### 2.4. Eye Tracking Assessment and Oculomotor Parameters

Eye movement recordings were obtained using a binocular eye tracking system according to a standardized study protocol. Exported eye tracking data were processed to derive fixation, saccadic, and binocular coordination parameters. The main oculomotor outcomes included fixation duration, microsaccade frequency, saccade frequency, saccade amplitude, peak velocity, latency, vergence proxy, binocular synchrony, fixation disparity, and conjugacy. These parameters were selected to reflect fixation control, saccadic dynamics, and binocular coordination.

### 2.5. Parental Questionnaire Variables

Questionnaire data included paternal and maternal education categories and parental stress or sleep status. Because the rationale for a direct association between parental education style and eye movement parameters was considered exploratory, these variables were analyzed as secondary factors and interpreted cautiously.

### 2.6. Statistical Analysis

Continuous variables are presented as mean ± standard deviation or median with interquartile range, as appropriate. Categorical variables are presented as counts and percentages. Three group comparisons were performed using Kruskal–Wallis tests for continuous variables and chi-square or Fisher exact tests for categorical variables. Oculomotor parameters were compared among the three lens groups using Kruskal–Wallis tests because several eye movement variables were non-normally distributed. Pairwise comparisons were performed using Wilcoxon rank sum tests with Benjamini–Hochberg adjustment.

Multivariable linear regression models were used to evaluate associations between lens group and each oculomotor parameter after adjustment for age, sex, baseline spherical equivalent, and baseline axial length. Spearman correlation and adjusted linear regression were used as exploratory analyses to examine the relationship between selected oculomotor parameters and axial elongation. One-year spherical equivalent change was compared between the defocus spectacle and diffusion optics technology groups using the Mann–Whitney U test. Spearman correlation was also used to examine the association between spherical equivalent change and selected oculomotor parameters in the two spectacle lens groups. False discovery rate correction was applied to account for multiple comparisons. Statistical analyses were performed using R software version 4.5.3 (R Foundation for Statistical Computing, Vienna, Austria). A two-sided *p* value < 0.05 was considered nominally significant. FDR adjusted *p* values were used to interpret multiplicity-adjusted findings.

## 3. Results

### 3.1. Baseline Characteristics

A total of 134 children were included, comprising 46 in the orthokeratology group, 49 in the defocus spectacle group, and 39 in the diffusion optics technology group. The orthokeratology group was older and had greater baseline myopia and longer baseline axial length than the two spectacle lens groups. Sex distribution was broadly similar across groups. Baseline characteristics are summarized in Table 1.

### 3.2. Group Differences in Oculomotor Parameters

**Figure 1:**
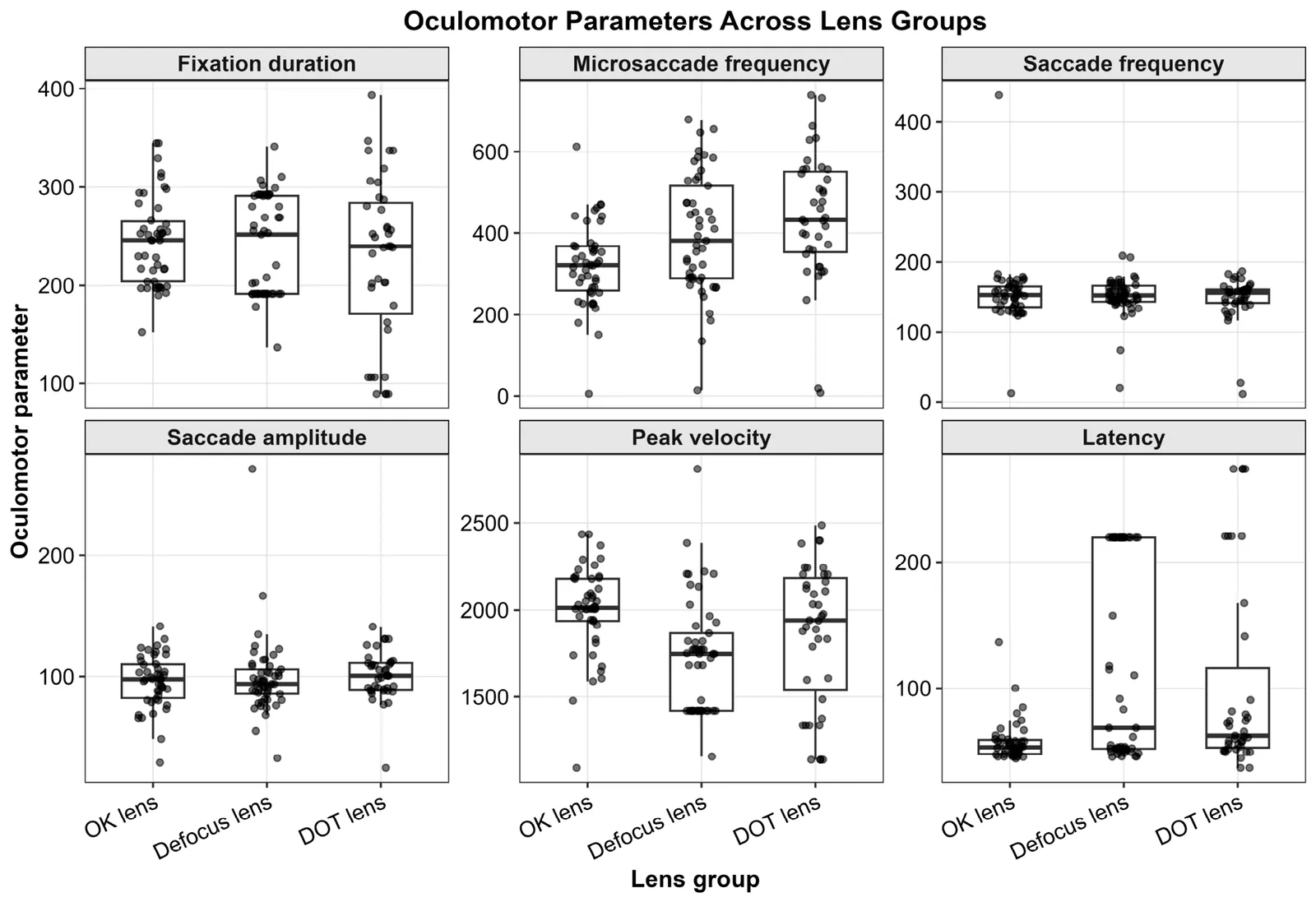
Oculomotor parameters across lens groups. Boxplots and individual data points show fixation duration, latency, microsaccade frequency, peak velocity, saccade amplitude, and saccade frequency across orthokeratology, defocus spectacle, and diffusion optics technology lens groups.

Kruskal–Wallis tests showed significant differences among lens groups in microsaccade frequency (FDR adjusted *p* = 0.00099), peak velocity (FDR adjusted *p* = 0.00099), latency (FDR adjusted *p* = 0.00395), fixation disparity (FDR adjusted *p* = 0.00395), and conjugacy (FDR adjusted *p* = 0.0274). Fixation duration, saccade frequency, saccade amplitude, vergence proxy, and binocular synchrony did not differ significantly among groups after FDR correction. Descriptive statistics and group-level test results are shown in Table 2 and Figure 1.

### 3.3. Pairwise Comparisons

Pairwise comparisons indicated that microsaccade frequency was higher in the defocus spectacle and diffusion optics technology groups than in the orthokeratology group. Peak velocity was significantly lower in the defocus spectacle group than in the orthokeratology group. Latency was significantly shorter in the orthokeratology group than in both spectacle lens groups. Fixation disparity differed between orthokeratology and defocus spectacle lenses and between defocus spectacle and diffusion optics technology lenses. Conjugacy differed between defocus spectacle and diffusion optics technology lenses (Table 3).

### 3.4. Adjusted and Sensitivity Analyses

In multivariable models adjusted for age, sex, baseline spherical equivalent, and baseline axial length, most lens group associations were attenuated after FDR correction. Defocus spectacle lenses showed nominally lower peak velocity compared with orthokeratology lenses (β = −252.9 deg/s, nominal *p* = 0.040), but this association did not remain significant after FDR correction. Because the orthokeratology group was older than the two spectacle lens groups, age was examined specifically. Age showed a negative correlation with microsaccade frequency and latency, indicating that age-related differences may have contributed to the unadjusted group findings (Table 4).

One-year axial elongation did not differ significantly among the three lens groups. Microsaccade frequency was correlated with axial elongation in unadjusted analysis, but the association was attenuated in the fully adjusted model. In the analysis limited to the two spectacle lens groups with available refractive follow up, one-year spherical equivalent change did not differ significantly between the defocus spectacle and diffusion optics technology groups (Table 5). The mean spherical equivalent change was −0.27 ± 0.43 D in the defocus spectacle group and −0.34 ± 0.36 D in the diffusion optics technology group (*p* = 0.312). No statistically significant correlation was observed between spherical equivalent change and microsaccade frequency in the spectacle lens groups (Spearman *r* = −0.231, *p* = 0.069). These findings do not support a direct interpretation of the observed oculomotor differences as evidence of different refractive progression between the two spectacle lens groups.

### 3.5. Exploratory Parental Variables

Parental education categories were analyzed as exploratory secondary variables. Paternal education category 2 showed nominal associations with fixation duration (β = −41.9 ms, *p* = 0.005), peak velocity (β = −178.6 deg/s, *p* = 0.024), and latency (β = 39.6 ms, *p* = 0.015), but none remained significant after FDR correction. Maternal education category was not significantly associated with oculomotor parameters after adjustment. Selected model estimates are shown in Table 6.

## 4. Discussion

In this real-world observational study of 134 children undergoing pediatric myopia management, different oculomotor profiles were observed among orthokeratology, defocus spectacle, and diffusion optics technology lens groups. After FDR correction, group differences were found in microsaccade frequency, peak velocity, latency, fixation disparity, and conjugacy. In contrast, fixation duration, saccade frequency, saccade amplitude, vergence proxy, and binocular synchrony did not differ significantly. These results suggest selective differences in fixation micromovements, saccadic dynamics, and selected binocular coordination measures rather than a broad difference in overall eye movement behavior.

The clinical meaning of oculomotor differences in myopia management should be interpreted cautiously. Axial length and spherical equivalent remain the established clinical outcomes for evaluating myopia progression and treatment response [3,4,5]. Eye tracking parameters are not validated substitutes for these outcomes. In the present study, axial elongation did not differ significantly among lens groups, and the correlation between microsaccade frequency and axial elongation was not robust after adjustment for age, sex, baseline spherical equivalent, baseline axial length, and lens group. In addition, one-year spherical equivalent change did not differ significantly between the defocus spectacle and diffusion optics technology groups. These results indicate that the observed oculomotor differences should be interpreted as visual behavior and adaptation-related findings, not as evidence that one lens type produced better myopia control.

The additional refractive change analysis was included to address the relationship between eye movement measures and refractive progression. Spherical equivalent change was examined only in the two spectacle lens groups because orthokeratology can alter post-treatment refraction through corneal reshaping. The absence of a significant difference in spherical equivalent change between the defocus spectacle and diffusion optics technology groups suggests that the higher microsaccade frequency and the binocular coordination differences were not accompanied by a detectable difference in one-year refractive progression in the available spectacle lens data. This finding supports a conservative interpretation of the eye tracking results and emphasizes that longitudinal studies with standardized cycloplegic refraction and axial length measurements are needed.

The age imbalance among lens groups is an important consideration. Children in the orthokeratology group were older and had greater baseline myopia and longer baseline axial length than children in the spectacle lens groups. Developmental eye movement studies have shown that saccadic latency can decrease with age, whereas peak velocity may be more stable or more dependent on saccade amplitude [13,14,15]. In this dataset, age was correlated with microsaccade frequency and latency. Therefore, age may have contributed to some unadjusted group differences. Although adjusted models were used, residual developmental effects cannot be excluded. The findings should, therefore, be viewed as descriptive real-world associations rather than causal effects of lens type.

The higher microsaccade frequency observed in the spectacle lens groups may reflect differences in fixation control, visual sampling, or attention-related processes during viewing through optical designs that modify retinal defocus or contrast. Microsaccades have been linked to fixation maintenance and covert attentional processes [16,17,18]. Diffusion optics technology lenses are intended to modulate retinal contrast, and recent clinical and optical evidence suggests that myopia control spectacles can differ in contrast reduction profiles [9,10,11]. However, the present design cannot determine whether microsaccade differences were caused by optical design, baseline group characteristics, task-related attention, or individual adaptation.

Latency was shorter in the orthokeratology group than in both spectacle lens groups in unadjusted pairwise comparisons. Saccadic latency reflects visual detection, attentional allocation, and motor preparation. Daytime unaided viewing after overnight orthokeratology differs from daytime viewing through spectacle lens structures, but the observed latency difference cannot be interpreted as functional superiority of orthokeratology. The association was sensitive to age and baseline ocular differences, and pretreatment eye tracking data were not available.

Peak velocity was lower in the defocus spectacle group than in the orthokeratology group in pairwise comparisons. Peak velocity is related to saccade amplitude and the main sequence properties of the saccadic system. Because saccade amplitude did not differ significantly among groups, the velocity finding may indicate subtle differences in motor execution or visual guidance. However, the adjusted model did not remain significant after FDR correction. This supports a cautious interpretation.

Fixation disparity and conjugacy differed among lens groups, whereas vergence proxy and binocular synchrony did not. These mixed results suggest selective differences in binocular coordination measures rather than generalized binocular dysfunction. Previous reports have drawn attention to the possible relationship between binocular visual function and axial growth [19,20]. The present results are consistent with the broader concept that binocular coordination may be relevant to pediatric visual assessment, but they do not show that any lens type impairs binocular vision or provides superior binocular function.

The parental education findings were intentionally reframed as exploratory. Family factors may influence treatment choice, adherence, visual habits, outdoor activity, and follow-up behavior, and educational interventions may improve parent knowledge and some eye health behaviors [21,22]. However, a direct link between parental education style and short-term oculomotor parameters has not been established. In this dataset, parental education categories were not robustly associated with oculomotor outcomes after FDR correction. These results should not be used to assign responsibility to caregivers or to guide lens choice.

This study has several strengths. First, three commonly used myopia control lens modalities were evaluated in a routine pediatric clinical setting. Second, multiple oculomotor parameters were analyzed, allowing fixation-, saccadic-, and binocular coordination-related measures to be described together. Third, FDR correction and multivariable adjustment were applied, and additional sensitivity analyses were performed to address age imbalance and the possible relationship with axial elongation and refractive change.

Several limitations should be acknowledged. First, the study was observational, and lens selection was not randomized. Residual confounding and selection bias remain possible despite adjusted analyses. Second, pretreatment eye tracking data were unavailable, so changes in oculomotor parameters after lens initiation could not be evaluated. Third, the three lens groups differed in age, baseline spherical equivalent, and baseline axial length. Fourth, axial elongation and spherical equivalent change were examined only as exploratory outcomes, and the study was not designed or powered to determine whether eye movement parameters predict myopia progression. Fifth, spherical equivalent change was not analyzed in the orthokeratology group because post-treatment refraction may be affected by corneal reshaping. Sixth, parental education categories were based on questionnaire coding and may not fully represent parenting style, socioeconomic status, or the home visual environment. Seventh, eye tracking was performed under a specific testing protocol, and the findings may not generalize to reading, classroom learning, outdoor activity, or other real-life visual tasks.

## 5. Conclusions

Children wearing orthokeratology, defocus spectacle, and diffusion optics technology lenses showed selective differences in oculomotor profiles, particularly in microsaccade frequency, peak velocity, latency, fixation disparity, and conjugacy. One-year spherical equivalent change did not differ significantly between the defocus spectacle and diffusion optics technology groups. The observed oculomotor differences should not be used to judge the superiority of one lens type or to replace axial length and refractive outcomes in the evaluation of myopia control efficacy. In clinical practice, eye tracking may be considered as an additional assessment when children report visual discomfort, adaptation difficulty, or binocular visual symptoms during lens wear. Prospective studies with age balanced or matched designs, baseline eye tracking, standardized visual tasks, and longitudinal axial length and refractive outcomes are recommended.

## Figures and Tables

**Table 1:** Baseline characteristics of participants.

Characteristic	OK Lens (*n* = 46)	Defocus Lens (*n* = 49)	DOT Lens (*n* = 39)	*p* Value
Age, years	12.11 ± 1.59	10.14 ± 1.63	9.44 ± 1.48	<0.001
Male, *n* (%)	25 (54.3)	28 (57.1)	20 (51.3)	0.912
Baseline SE, D	−2.90 ± 0.97	−1.31 ± 0.75	−1.75 ± 0.95	<0.001
Baseline AL, mm	24.79 ± 0.77	23.95 ± 0.88	23.89 ± 0.83	<0.001
Paternal education category 1, *n* (%)	30 (65.2)	33 (67.3)	26 (66.7)	0.730
Maternal education category 1, *n* (%)	41 (89.1)	43 (87.8)	31 (79.5)	0.851
Paternal stress or poor sleep, *n* (%)	15 (32.6)	20 (40.8)	15 (38.5)	0.700
Maternal stress or poor sleep, *n* (%)	13 (28.3)	23 (46.9)	15 (38.5)	0.172

Values are mean ± SD unless otherwise indicated. SE = spherical equivalent; AL = axial length. *p* values for age, SE, and AL were obtained using Kruskal–Wallis tests. *p* values for categorical variables were obtained using chi-square or Fisher exact tests.

**Table 2:** Oculomotor parameters across lens groups.

Oculomotor Parameter	OK Lens	Defocus Lens	DOT Lens	*p* Value	FDR Adjusted *p*
Fixation duration, ms	244.31 ± 44.97	239.16 ± 50.42	226.25 ± 83.71	0.632	0.702
Microsaccade frequency	321.30 ± 101.53	395.33 ± 146.12	438.15 ± 158.10	<0.001	<0.001
Saccade frequency	155.16 ± 50.30	151.61 ± 28.16	146.70 ± 34.19	0.931	0.931
Saccade amplitude, deg	95.99 ± 22.01	98.79 ± 32.69	100.86 ± 20.28	0.316	0.452
Peak velocity, deg/s	2000.11 ± 255.00	1727.37 ± 327.98	1861.95 ± 403.20	<0.001	<0.001
Latency, ms	58.01 ± 16.35	117.78 ± 78.01	103.36 ± 78.95	0.001	0.004
Vergence proxy	0.24 ± 0.16	0.23 ± 0.15	0.26 ± 0.16	0.592	0.702
Binocular synchrony	0.63 ± 0.20	0.62 ± 0.18	0.69 ± 0.17	0.196	0.327
Fixation disparity	−0.02 ± 0.25	−0.10 ± 0.21	0.05 ± 0.20	0.002	0.004
Conjugacy	0.02 ± 0.01	0.01 ± 0.01	0.02 ± 0.01	0.014	0.027

Values are mean ± SD. *p* values were obtained using Kruskal–Wallis tests. FDR = false discovery rate.

**Table 3:** Pairwise comparisons for oculomotor parameters showing significant overall group differences.

Parameter	Comparison	*p* Value	Adjusted *p*
Microsaccade frequency	OK lens vs. Defocus lens	0.010	0.015
Microsaccade frequency	OK lens vs. DOT lens	<0.001	<0.001
Microsaccade frequency	Defocus lens vs. DOT lens	0.098	0.098
Peak velocity, deg/s	OK lens vs. Defocus lens	<0.001	<0.001
Peak velocity, deg/s	OK lens vs. DOT lens	0.222	0.222
Peak velocity, deg/s	Defocus lens vs. DOT lens	0.052	0.079
Latency, ms	OK lens vs. Defocus lens	0.002	0.003
Latency, ms	OK lens vs. DOT lens	0.001	0.003
Latency, ms	Defocus lens vs. DOT lens	0.787	0.787
Fixation disparity	OK lens vs. Defocus lens	0.012	0.018
Fixation disparity	OK lens vs. DOT lens	0.165	0.165
Fixation disparity	Defocus lens vs. DOT lens	<0.001	0.003
Conjugacy	OK lens vs. Defocus lens	0.059	0.089
Conjugacy	OK lens vs. DOT lens	0.656	0.656
Conjugacy	Defocus lens vs. DOT lens	0.002	0.007

Pairwise comparisons used Wilcoxon rank sum tests with Benjamini–Hochberg adjustment within each parameter.

**Table 4:** Exploratory sensitivity analyses addressing age, axial elongation, and refractive change.

Analysis	Parameter	Estimate	*p* Value	FDR Adjusted *p*
Age correlation	Microsaccade frequency	Spearman *r* = −0.523	<0.001	<0.001
Age correlation	Latency	Spearman *r* = −0.264	0.002	0.010
Axial elongation by lens group	Mean one-year axial elongation	Kruskal–Wallis H = 2.013	0.365	Not applied
Axial elongation correlation	Microsaccade frequency	Spearman *r* = 0.285	<0.001	0.008
Fully adjusted axial elongation model	Microsaccade frequency	β = 0.0002 mm per unit	0.092	0.460
Refractive change comparison	Mean one-year SE change in defocus vs. DOT groups	Mann–Whitney U = 566.5	0.312	Not applied
Refractive change correlation	SE change and microsaccade frequency in spectacle lens groups	Spearman *r* = −0.231	0.069	Not applied

Axial elongation was calculated as one-year mean axial length minus baseline mean axial length. Spherical equivalent change was calculated as one-year mean spherical equivalent minus baseline mean spherical equivalent and was analyzed only in the defocus spectacle and diffusion optics technology groups. Negative spherical equivalent change indicates myopic shift. Fully adjusted axial elongation models included age, sex, baseline spherical equivalent, baseline axial length, and lens group.

**Table 5:** One-year spherical equivalent change in the two spectacle lens groups.

Group	*n*	Mean ± SD, D	Median (IQR), D	*p* Value
Defocus lens	34	−0.27 ± 0.43	−0.19 (−0.50 to 0.00)	0.312
DOT lens	29	−0.34 ± 0.36	−0.31 (−0.56 to 0.00)	

Values are presented as mean ± SD and median with interquartile range. *p* value was obtained using the Mann–Whitney U test. The orthokeratology group was not included because post-treatment refraction may be affected by corneal reshaping.

**Table 6:** Adjusted associations between parental education categories and selected oculomotor parameters.

Exposure	Outcome	Estimate	*p* Value	FDR Adjusted *p*
Paternal education category 2 vs. 1	Fixation duration, ms	β = −41.92	0.005	0.122
Paternal education category 2 vs. 1	Microsaccade frequency	β = −3.03	0.925	0.991
Paternal education category 2 vs. 1	Saccade frequency	β = −6.11	0.411	0.765
Paternal education category 2 vs. 1	Saccade amplitude, deg	β = 5.95	0.390	0.765
Paternal education category 2 vs. 1	Peak velocity, deg/s	β = −178.55	0.024	0.300
Paternal education category 2 vs. 1	Latency, ms	β = 39.59	0.015	0.241
Maternal education category 2 vs. 1	Fixation duration, ms	β = −2.99	0.886	0.977
Maternal education category 2 vs. 1	Microsaccade frequency	β = 58.82	0.184	0.590
Maternal education category 2 vs. 1	Saccade frequency	β = 11.82	0.248	0.639
Maternal education category 2 vs. 1	Saccade amplitude, deg	β = 13.72	0.149	0.590
Maternal education category 2 vs. 1	Peak velocity, deg/s	β = 48.28	0.664	0.916
Maternal education category 2 vs. 1	Latency, ms	β = −14.83	0.516	0.861

Models were adjusted for lens group, age, sex, baseline spherical equivalent, and baseline axial length. Category 1 was the reference category.

## Data Availability

The datasets generated and analyzed during the current study are available from the corresponding author on reasonable request, subject to institutional and ethical restrictions.
